# Skilled Action and the Double Life of Intention†

**DOI:** 10.1111/phpr.12433

**Published:** 2017-07-12

**Authors:** Joshua Shepherd

**Affiliations:** ^1^ University of Oxford

## 1 The Interface Problem

In bodily intentional action, an agent exercises control over her bodily behavior. An important part of the explanation of this involves a mental state of commitment to an action plan—that is, the agent's intention. The agent's intention (or its acquisition) initiates the action, and the continuance of the intention throughout the unfolding action plays important causal roles in sustaining and guiding the action to completion. But the agent's intention is not the only mental state operative in bodily intentional action. Recent work has emphasized important roles for lower‐level states as well: so‐called motor representations (Decety et al. 1994, Pacherie 2008). These lower‐level states specify movement details and movement outcomes in ways that respect fine‐grained biomechanical and temporal constraints upon intention satisfaction.

Butterfill and Sinigaglia (2014) have argued that in so doing motor representations are far from “philosophically irrelevant enabling conditions” (120). Rather, motor representations ‘ground the directedness of actions to outcomes’ (124). But, according to Butterfill and Sinigaglia, it is not clear how they do so. For they argue that intentions and motor representations have different representational formats. Intentions have a propositional format, and as such integrate with states and processes involved in practical reasoning. Motor representations have a “distinctively motor, non‐propositional format” (120). This generates a problem. Butterfill and Sinigaglia explain:

There are cases in which a particular action is guided both by one or more intentions and by one or more motor representations. In at least some such cases, the outcomes specified by the intentions match the outcomes specified by the motor representations. Furthermore, this match is not always accidental. How do non‐accidental matches come about? (131–132)

Butterfill and Sinigaglia call this *The Interface Problem*. To get a more vivid feel both for the problem and the interest inherent in it, consider the following study involving expert typists. Logan and Crump (2010) had skilled typists (average words per minute: 68) type four letter words under various conditions. In one condition, participants were cued to type either the whole word, or only to type the letters normally typed by the left or right hand. Unsurprisingly, performance speed and accuracy were extremely degraded when typing the words required inhibition of certain keystrokes. This result seems to be driven by the requirement that the typists extract the keystrokes made by one hand and use only those. This is very difficult because the processes that assemble and sequence whole‐word keystroke patterns are relatively inaccessible: the typists do not know “which hand types which letters” (Logan and Crump 2011, 13). Although the typists can easily and quickly type a word, as they do so they have little understanding of how their sequence of movements is constructed and implemented. This suggests that although proposition‐level action understanding guides the sequencing of the action at some level (i.e., at the level of word construction), this level of understanding is out of touch with the unfolding of the action at other levels (i.e., the level of finely sequenced keystrokes). But of course skilled actions like typing are performed fluidly, and skilled typists display a fine‐grained sensitivity to errors even at the level of the individual keystroke. How does propositional‐level action understanding coordinate with motoric‐level action implementation to produce such behavior?

Given that a solution to the interface problem will involve a more satisfying explanation of the respective contributions of propositional‐level and motoric‐level processing, the solution will be of more than intrinsic interest. In addition, it is likely to have downstream theoretical consequences. A number of interesting recent debates cluster around the interface between these aspects of action control.

Stanley and Krakauer (2013) argue that “performing a skilled motor action in *any ordinary sense* (where the paradigm cases are activities like tennis, cooking, dancing) centrally involves propositional knowledge” (8). Of course these actions require certain kinds of motor abilities as well, but Stanley and Krakauer place the role of intelligence and knowledge entirely at the propositional level. Motoric‐level learning contributes to motor acuity, that is, “practice‐related reductions in movement variability and increases in movement smoothness” (8), but in a way that is not directly knowledge involving. Rather, in skilled action “the musician or athlete is using knowledge of the musical score or the game to dictate to those automatic non‐knowledge based components; it is the combination that leads to the skilled performance” (10).

An implication of this view is that the achievements subserved by motoric‐level processing are beyond the scope of rational appraisal. Luthra argues explicitly that motoric‐level abilities are not open to rational appraisal, claiming that “our agency, our control over what we do, consists partly in non‐rational action guiding capacities” (2016, 2268).

By contrast, a number of philosophers have in various ways taken an opposing view regarding motoric‐level processing. Neil Levy (2017) argues that although motor representations are not propositionally structured, they operate in intelligent processes—i.e., processes that “flexibly adapt in an appropriate manner to environmental perturbations” (517)—and they “are representations in virtue of which agents possess knowledge” (522). Ellen Fridland (2017) argues that “the motor control involved in skill is intelligent all the way down” (1540), where intelligence for Fridland means roughly what it does for Levy, and implies that motor control is more than “a brute‐causal, bottom‐up system that becomes tuned through simple repetition” (1557). And Chiara Brozzo (2017) argues that some motor representations qualify as motor *intentions*, and as such are open to rational appraisal.

How does the interface problem relate to disputes about the intelligence or rationality of motoric‐level processing, or its relation to knowledge? Given the experimental evidence that propositional‐level action understanding and motoric‐level action implementation do, in some cases, come apart, we need a better understanding of the manifest fact that in most cases they seem to work together. Without this, our understanding of the nature of skilled action and of the scope of practical rationality remains truncated. Consider, for example, the skilled typist. Her propositional‐level action understanding coordinates in fluid, fine‐grained ways with motoric‐level action implementation. Notice that without a solution to the interface problem, the view that motoric‐level processing is intelligent, knowledge‐involving, or open to rational appraisal risks commitment to something like two centers of agency present in the skilled typist (and in many bodily actions)—the center governed by propositional‐level understanding of the action at hand, and the center governed by motoric‐level understanding of the action at hand. If the latter operate in an intelligent way, then our picture of the skilled typist is of two distinct systems operating intelligently on different aspects of the action, somehow managing to generate coordinated control of the same action. Even if human action control runs via two distinct and distinctly intelligent systems, we seem to need an explanation of how these systems manage to interface and coordinate rather than to compete for the control of action.

The above authors recognize this, with Levy (2017) and Brozzo (2017) both explicitly endorsing Butterfill and Sinigaglia's (2014) proposal (which I reject below). And Fridland closes her paper by discussing the interface problem, noting—quite plausibly—that “an adequate account of skill will require a substantive account of control not only at the intentional and motor level, but also a robust theory of the integration between the two” (1558).

In what follows I propose a solution to the interface problem. Before I do, I discuss two proposals currently afloat, due to Butterfill and Sinigaglia (2014), and to Mylopoulos and Pacherie (2017). For reasons I discuss, neither proposal is fully satisfying. In particular, both proposals seek to avoid commitment to a ‘translation process’ between intentions and motor commands. By contrast, I think a focus on a translation process may be just what we need. Building on recent experimental results, I argue that with respect to representational format, intentions lead a double life. Intentions can take propositional or motoric formats, and proximal intentions sometimes take both. The agent's capacity to put these formats together in rational ways explains the non‐accidental link between proposition‐level understanding of action, and motoric‐level implementation of action.

## 2 Previous Proposals

Butterfill and Sinigaglia's proposed solution to the interface problem involves demonstrative and deferential action concepts. They illustrate these notions by discussing the relation between a cartographic and a propositional representation of a route. The propositional representation demonstratively refers to the cartographic representation via the sentence ‘Follow this route!’ So the propositional representation defers to the cartographic representation. According to Butterfill and Sinigaglia, “Because the representation deferred to is cartographic, comparing the instruction with the map no longer requires translation between representational formats” (133). The same relationship holds, they claim, between intention and motor representation.

This absence of a translational process is important to Butterfill and Sinigaglia. They reject any proposed solution that requires a translation process between proposition‐level action understanding and motoric‐level action implementation because “nothing at all is known about this hypothetical translation between intention and motor representation, nor about how it might be achieved, nor even about how it might be investigated” (133). According to Butterfill and Sinigaglia, their proposal skirts this worry because no process of translation is needed between intention and motor representation. How, then, does intention manage to demonstratively connect with motor representation? Butterfill and Sinigaglia's explanation begins with the claim that motor representations “are available in some sense” (134). More specifically, they claim that certain kinds of conscious experiences—those associated with imagining acting and actually acting—are required for development of the demonstrative component of intention. Returning to the cartographic analogy, they write:

Someone encounters a map with a route marked on it. Her experience of this route is necessary for her to acquire a demonstrative concept which refers to the route by deferring to the cartographic representation of it. But once she has this demonstrative concept, she can use it on future occasions without fresh experiences of the route (although there may be some dependence on memory); and her use of this concept does not depend on the continued existence of the original representation of the route. Similarly, on our view experience of action is necessary for the acquisition of demonstrative concepts of action such as concepts of grasping and reaching but, perhaps subject to requirements on memory, not for their continued use in practical thought. (135)

The picture they give us, then, is that proposition‐level action understanding interfaces with motoric‐level action implementation via a demonstrative component of an action concept within an agent's intentions. In demonstrating, this component defers to the motor representation, which thereby determines the content of the intention.

Although I like this proposal, I think it cannot succeed in full generality. In a recent paper, Mylopoulos and Pacherie (2017) offer one compelling reason why. They argue that far from avoiding a translation process, Butterfill and Sinigaglia's proposal presupposes one:

[I]n the case of demonstrative deferral in intention, the agent must have an independent grasp of which motor representation is the appropriate one to select via such deferral. But this would require a way of translating between the intention and the motor representation being picked out, in order to establish which motor representation correctly corresponds, and Butterfill and Sinigaglia have already argued that we know nothing about how this translation process works. (329)

The charge is that the notion of demonstrative deferral at issue smuggles in something Butterfill and Sinigaglia do not explain: how the intention manages to defer to the right motor representation without any translation process. A further worry relevant in this connection is that the process of deferral remains opaque. Butterfill and Sinigaglia attempt to illuminate deferral with their discussion of demonstration, but claim in the end that demonstration is not essential: “what matters for solving the interface problem is deference, not demonstration” (140). But they say nothing about deferral apart from an example involving an action of demonstration. What are we to think of it? At this point one may be tempted to share Mylopoulos and Pacherie's skepticism “that this putative psychological phenomenon occurs” (328).

Mylopoulos and Pacherie have a counter‐proposal. They offer a version of the content‐preserving causal process solution that Butterfill and Sinigaglia rejected due to worries about translation. Importantly, however, they deny that their proposal involves “a mysterious translation process” (325). How does it work?

As a part of their proposal, Mylopoulos and Pacherie introduce the notion of an executable action concept. This is a concept of an action in virtue of which it can be thought and reasoned about. Furthermore, in being executable, it is a concept of an action that an agent has the ability to perform. According to Mylopoulos and Pacherie, possession of such a concept depends upon possession of a motor schema, which is related to but more abstract and stable than the motor representations involved in action execution.

[T]he motor representations that guide specific actions are instantiations of motor schemas where the values of the parameters that control the action are specified and then updated depending on sensory information and feedback. Motor schemas are thus more abstract and enduring representations of actions that store knowledge about the invariant aspects and the general form of an action. (330–331)

Mylopoulos and Pacherie make a convincing case that agents come to possess motor schemata by way of Bayesian learning. It follows, then, that as an agent acquires competence with respect to some action‐type, she will develop relevant motor schemata, which will play a role—Mylopoulos and Pacherie are not explicit about what role, beyond the dependence relation already elucidated—in her possession of an executable action concept. Presumably, once she is so situated, an agent will be able to slot an executable action concept into an intention, and the connection between the concept and the motor schema will link proposition‐level understanding to motoric‐level implementation.

At this point, however, one wants to know more about the relationship between the action concept and the motor schema. How does the deployment of an action concept link up with a motor schema? That they call an action concept a concept, and aver that agents can think and reason about their actions in virtue of such concepts, suggests it engages with processes at the propositional level. And that a motor schema is an abstraction from motor representations suggests it is coded in a motoric representational format. But this is just the joint that generated the interface problem. For all Mylopoulos and Pacherie say, we do not know how their contents are related. If agents cannot use the contents of motor schemata in proposition‐level practical reasoning—a possibility suggested by Mylopoulos and Pacherie's claim that motor representations are inaccessible to consciousness – then one wonders how, after all, action concepts and motor schemata non‐accidentally link up in action control.

Mylopoulos and Pacherie's proposal does not end with the positing of action concepts and motor schemata. They note that in addition to these two elements, action execution “also demands that the information needed to set the value of the schema's parameters be selected and encoded in a format readily exploitable by the motor system” (331). Here, however, it seems we need the translation process Mylopoulos and Pacherie denied needing.^2^ For this is just the place at which the interface problem arises. How does one's propositional‐level understanding of an action link up with the motoric‐level processing that executes intentions? Mylopoulos and Pacherie invoke a selective role for attention in setting schema parameters, but this looks like redescription rather than explanation. We can agree that some selection process will be important in setting parameters. The question is how this process could move from the information at the propositional‐level of action understanding to information at the motoric‐level of action implementation. It looks like we still do not know.^3^

## 3 Relevant Experimental Results

Recent experimental results light the way to a solution. The results stem from experiments utilizing a visuomotor rotation task. In this task participants see targets on a screen, sometimes flanked by aiming landmarks around a circle. They cannot see their hand, but they can see a cursor on a screen. The cursor represents movements of a hand‐controlled stylus towards targets displayed on the screen. The use of the screen and the cursor allows experimenters to manipulate the visual feedback participants receive. In standard visuomotor rotation experiments, for example, experimenters will present the cursor as moving at a 45 degree angle away from where the hand is actually moving. This allows them to see how the sensorimotor system adapts to this unexpected feedback. Typically, participants display drift in the direction opposite the visuomotor rotation. That is, they display implicit learning that in order to hit the target they need to correct for the gap between their aim and the visual feedback they receive. With appropriate manipulations the task can be very informative about basic principles of sensorimotor learning and control.

One interesting development in research on sensorimotor adaptation is a growing appreciation on the part of cognitive scientists that what they call strategy use—i.e., practical reasoning about features of the task, and the formation of conscious intentions based on such reasoning—is an important part of even basic sensorimotor adaptation and control. This is a recent development because, as McDougle et al. explain in a recent review paper:

Until recently, strategy use has been considered a nuisance in studies of sensorimotor adaptation, and experimental instructions are often designed to actively discourage this behavior. Moreover, the use of heuristics, such as an explicit change in aiming, has been ignored in computational models of the learning process. (2016, 536)

A number of studies have shown, however, that the use of explicit strategies is important for sensorimotor adaptation in a few ways. For example, after allowing participants to perform a few reaching actions under rotated (i.e., non‐veridical) visual feedback, Mazzoni and Krakauer (2006) interrupted participants and told them about the rotated feedback. (Participants did not receive continuous feedback in this study: only feedback about the outcome. This makes it more difficult for them to get an immediate sense that something is awry.) They also facilitated the use of an explicit aiming strategy by positioning potential targets around the visual array at 45 degree increments. Participants immediately corrected for the rotation thanks to their explicit strategy. This is what cognitive scientists would call ‘one‐trial learning’ (McDougle et al. 2016, 536). Surprisingly, however, after this one‐trial learning effect participants began to show drift. Even though they explicitly knew they had corrected for the visuomotor rotation, sensorimotor adaptation processes began to drive their reaching actions away from the location of aim in accordance with standard monotonic updating in response to the visual feedback.

Taylor and Ivry (2011) replicated this finding, but gave participants an increased number of trials: 320, compared with 80 in the Mazzoni and Krakauer study. With an increased number of trials, participants had time to counteract the drift produced by implicit learning. By the end of the trial block, participant error had been almost completely eliminated. Taylor and Ivry note that in a debriefing session, some participants noted their explicit change in aiming strategy to counteract the effects of implicit learning.

These results already challenge any model on which intentions relate to motor representations by a process of deferral. For the participants in these experiments do not defer to the motor representations in question. They use their knowledge of how the motor adaptation processes are functioning to *override* them.

These results, along with nearby results in the sensorimotor learning literature, allow further characterization of the gap between proposition‐level action understanding and motoric‐level action implementation. The gap can be characterized in part by the kind of signals to which these levels are sensitive. As McDougle et al. (2016) explain, implicit learning appears primarily sensitive to sensory prediction error—the mismatch between expected and observed sensory feedback. But explicit processes are sensitive to performance error—the mismatch between the explicit goal (i.e., the relationship between the location at which an agent consciously aims and the target an agent intends to hit) and the observed outcome. In the Mazzoni and Krakauer (2006) and Taylor and Ivry (2011) studies, the implicit processes continue to drive drift in spite of action success because “implicit recalibration is completely insensitive to task success” (McDougle et al. 2016, 539).^4^

The dissociation between explicit and implicit processes these studies uncover can drive theoretical attention to the distance between them. But it is important to remember that everyday action success, and indeed the ability of participants to correct for unwanted implicit learning in these studies, demonstrates that these processes almost always somehow work together to coordinate action. How this happens is, of course, what the interface problem challenges us to explain. In this connection, I find a very recent study by Day et al. (2016) illuminating.

Day et al. utilized the visuomotor rotation paradigm, with a few tweaks. First, following Bond and Taylor (2015), they had participants report their aiming location before each movement. This allowed better measurement of the relation between explicit aiming strategies and implicit learning, as we will see. Second, they interspersed ‘catch trials’ throughout the normal trials. During a catch trial, participants were instructed to aim directly at a target with all other aiming landmarks and visual feedback removed. This allowed a direct measurement of ‘implicit learning’—the difference in location between the actual movement and the aimed‐at target. Third, they changed the direction of the aiming location throughout these catch trials for different sets of participants. More specifically, they had some participants aim to commonly aimed‐at locations, and they had other participants aim to novel locations.

Day et al. found the occurrence of implicit learning throughout the task. That is, participants consistently moved farther away from their reported aiming location in a direction suggestive of implicit learning. Very interestingly, however, they observed differences in the amount of implicit learning based upon the direction of a participant's reach. They explain these differences as follows.

[A]s participants aimed farther from their most frequently reported aiming location, the magnitude of implicit learning decreased. Thus, implicit learning generalized maximally at each individual's most frequent aiming location and decays as a function of angle away from that aiming location. (7)

That implicit learning generalized around the location of aim and not the location of actual movement is significant. One clear inference to make, and which Day et al. make, is this.

There is obvious interplay between the cognitive and implicit processes involved in motor adaptation… the two are not merely engaged in a simple give‐and‐take relationship to achieve task goals, but rather the implicit sensorimotor recalibration that defines visuomotor adaptation is learned around the cognitive representation of the movement. (11)

The relevance of the foregoing to the interface problem is probably apparent by now. Before addressing that, however, it may help to make explicit the general picture these results offer. At least four points are relevant.

First, there is a dissociation in the way proposition‐level action understanding and motoric‐level action implementation work towards goal achievement. This dissociation shows up in studies that separate feedback about action outcomes from sensory feedback. In the standard visuomotor rotation task, the participant aims to one place in order to hit a target at another place. If successful, she knows this and need not change her explicit strategy. But implicitly, her motor system will recalibrate based upon the difference between the aiming location and the visually presented target.

Second, and importantly, the best way to understand this dissociation is that the explicit aiming location not only represents the desired outcome at an explicit level, it also sets expectations at the implicit level. The conscious intention has a double life. It not only emanates from the agent's explicit use of practical reasoning, it directs aiming in real time, allowing one‐trial learning. And it drives sensorimotor adaptation, as it is the conscious intention that sets the location around which implicit learning occurs.^5^

Third, although sensorimotor adaptation takes information from the conscious intention as input, motoric‐level processes are not entirely passive. If sensory feedback is non‐veridical, these sensorimotor adaptation processes will tweak the conscious intention in order to minimize the sensory prediction error. So, over time, you can get the awkward situation in which an agent's intention to aim to location [x] ultimately results in an action of aiming to location [x as recalculated by implicit learning]. This result will be rare in the wild, since it depends upon the receipt of non‐veridical sensory feedback. But it is worth noting that it is consistent with a well‐confirmed picture of the cognitive architecture of action control, on which action control is subserved by hierarchical levels of processing. On this picture lower levels operate quasi‐autonomously over representations coded at finer levels of grain, and higher levels operate over more abstract representations (for discussion, see Grafton and Hamilton 2007, Logan and Crump 2011, Shepherd 2015). So in cases like the ones under discussion lower levels in the action control hierarchy are operating in ways that normally lead to action success, but that in these cases do not.

Fourth, although they may not discern this awkward situation in every case, over time agents will come to have a sense of it. At such times agents are able to make an explicit contribution to action control such that they override implicit learning to achieve explicitly represented goals.

## 4 Bridging the Interface

Recall that the interface problem was generated by Butterfill and Sinigaglia's claim that while intentions are represented in a propositional format, motor representations take a “distinctively motor, non‐propositional format” (2014, 120). The problem is magnified, of course, if intentions take an exclusively propositional format—a claim that seems implied by Butterfill and Sinigaglia's discussion of the problem. In light of the above results, however, it is dubious that we should accept this implication.

When discussing the difference between propositional and motoric formats, Butterfill and Sinigaglia compare two exercises of imagination. We are told to contrast a former basketball player imagining playing basketball, and a non‐player imagining playing basketball using only her cognitive appreciation of how one might play. The former player's imaginative exercise will depend upon those “bio‐mechanical, dynamical and postural constraints… closely related to those which govern actually performing such actions” (129–130). The non‐player's imaginative exercise needn't so depend: “a cognitive kind of imagining need not involve imagining an action unfolding in a way consistent with one's actual abilities” (130). Reflecting on this difference, Butterfill and Sinigaglia claim “motor representations differ in format from those involved in cognitive kinds of imagination, which are plausibly propositional” (130).

Nothing they have said rules out the possibility that in addition to taking propositionally formatted contents, intentions can take motorically formatted contents as well. Indeed, this is a plausible interpretation of what is happening when the former player imagines playing. She imagines performing a range of intentional actions, which involve intentions that incorporate both propositional and motoric contents.

The solution to the interface problem is that intentions lead a double life. Intentions can take propositionally formatted contents that enable their integration with propositional thought. And intentions have motorically formatted contents that communicate in a fairly direct way with the operations of motoric‐level action implementation.^6^ The interface problem is a problem about how the outcomes specified in intentions could guide and constrain the outcomes specified in motor representations. The answer is that intentions specify outcomes both propositionally and motorically. This is not, of course, to say that intentions specify outcomes at the finest possible grain. There is clearly room for the independent operation of sensorimotor adaptation processes. What we need to understand, however, is how intentions could provide guidance sufficient to render our common action successes non‐accidental. Intentions do this by specifying outcomes that motoric‐level action implementation processes take on board directly.

In saying this, I am not claiming that intentions necessarily or always include motoric components. Distal intentions—intentions to A later—primarily function to constrain planning processes, and as such have little need for on the ground specifications of how one ought to move. As agents develop, they may increasingly rely on propositionally structured thought to reason about the future. Furthermore, it may be the case that human agents develop cognitive shortcuts—ways of referring to motor schemata—that enable something much like Butterfill and Sinigaglia's posited deferral process. If an action concept such as GRASPING MY COFFEE MUG becomes linked with the relevant motor schemata via repeated tokening within very similar intentions, there may come a time when all I need to grasp my coffee mug successfully is the tokening of an abstract, propositionally represented intention (e.g., GRASP MY MUG!), which then defers successfully to the associated motor schemata. In such a case, the learning process that links the action concept with the motor schemata will explain the non‐accidental nature of the link. My proposal is simply that in virtue of an agent's cognitive combinatorial capacities, an intention can take both propositional and motoric contents. That this can and does happen is the best way to explain patterns of behavioral data emerging from research on action control and sensorimotor adaptation.

There will be objections to this proposal. In the next section I reply to a few. Doing so will provide the opportunity to further elucidate and clarify the proposal.

## 5 Objections and Replies

### Objection

Your proposal involves intentions formed at least sometimes on the basis of explicit practical reasoning taking contents specified in a motoric representational format. But motor representations are inaccessible to consciousness (i.e., to explicit practical reasoning). So this proposal cannot work.

### Reply

Most people who write about motor representations claim only that they are often inaccessible to consciousness. They often make this claim as though it is obvious, although it is not. I can agree that motor representations are often not accessed without agreeing that they are often inaccessible. Both positions are consistent with the data that leads many to make the claim about frequent inaccessibility. This is data to the effect that low‐level changes in action implementation often occur without the agent's awareness that they occur. Of course, if motor representations are sometimes accessible, then this is no real objection to my proposal. I have already noted that intentions for well‐learned actions may no longer need to specify motorically formatted contents. What this objection needs to offer is a reason to think that motor representations are always inaccessible. I'm not sure there is such a reason.

Mylopoulos and Pacherie (2017) are more explicit than most about the purported inaccessibility of motor representations. According to them:

Rather than being the inputs or the outputs of practical reasoning processes, they are the inputs and outputs of rapid sensorimotor computations. Rather than being subject to norms of practical reasoning, they are subject to a set of biomechanical constraints and motor rules. Rather than being personal‐level representations, they are subpersonal representations. Rather than functioning under conscious control, they function largely automatically. (327–328)

But what is the evidence for claiming these states are subpersonal? It cannot be that they have a different representational format involving specification of biomechanical properties. Conscious states take many different representational formats. It must be the thought that given the rapidity and fineness of grain at issue in sensorimotor processing, the kinds of computations they enter into are not the kinds of things conscious thought can influence.

I do not deny that in virtue of their speed and, perhaps, their fineness of grain, aspects of sensorimotor processing are inaccessible to conscious thought. This does not entail that motor representations are unavailable for the construction of intentions before action has begun. Nor does it entail that, given sufficient time to engage ongoing motoric‐level implementation processes, motor representations cannot be deployed as correctives over the course of an unfolding action. After all, many important aspects of many different action‐types involve temporal constraints lax enough for conscious thought to make a difference, a point I have discussed at length in other work (Shepherd 2015).

A further point to make in reply is that if motor representations were not consciously accessible, the large literature on motor imagery would seem misguided. Indeed, Brozzo (2017) appeals to motor imagery to argue that motor representations at certain levels of abstraction are consciously accessible. The study of motor imagery proceeds under the assumption that in imagining action agents activate motor representations, which thereby color their imagery in certain ways. One might claim that in motor imagery one does not access motor representations, but rather sensory imagery tied to the imagined movements. But this claim is no more warranted by the data than the claim that in motor imagery one accesses motor representations directly.

Finally, if motor representations are not consciously accessible, it is much harder to explain how agents update their intentions in a way that coordinates with the results of implicit learning. We have seen that agents are able to make an explicit contribution to action control such that they override implicit learning to achieve explicitly represented goals. Further, we have seen that sensorimotor adaptatation processes depend upon elements of the explicitly generated intention, such as the direction of aim. The best explanation of the sensitivity of sensorimotor adaptation to the conscious intention is that the intention specifies the aiming direction in a way the sensorimotor processes can understand—a way that enables the generation of sensory expectations that can be compared against sensory feedback to generate a sensory prediction error and begin the learning process. Since the sensorimotor adaptation processes operate on motor representations, it is plausible that components of the conscious intention such as the direction of aim are specified in a motoric format.^7^

### Objection

Doesn't this proposal simply push the interface problem back a level? You have intentions taking propositionally and motorically formatted contents. But even if these contents are present in the same intention, how do they interface? Isn't some kind of translation process required here as well?

### Reply

Let us pause over this difference in representational format for a minute. We have been told that intentions are propositionally formatted, while motor representations have a motoric format. What is involved in possessing this non‐propositional, motoric format? In the literature such claims seem primarily to imply two things. First, motor representations enter into the computations proprietary to sensorimotor control and learning. Second, given this, there are constraints on the kinds of contents motor representations can take. Unlike propositional representations, motor representations must respect biomechanical and temporal properties of bodily movement profiles if they are to play their computational role. To this it is sometimes added that given the fineness of grain at issue in some instances of sensorimotor control and learning, motor representations are specified in a grain finer than that of the propositions or concepts an agent possesses or grasps.

That motor representations enter into certain motoric‐level computations and possess fine‐grained contents does not entail that elements of motor representations will be unavailable for use within explicit practical reasoning. To illustrate, notice that the claims made above about motor representations are similar to claims often made about perceptual representations. Proponents of non‐conceptual perceptual content point, among other things, to the fineness of grain within perceptual experience, claiming that it outstrips the concepts we possess. To this one might add that unlike propositional representations, perceptual experience must respect certain biomechanical properties of the sensory transducers, e.g. the way transducers proprietary to different modalities are sensitive to differing stimulation profiles. Opponents of non‐conceptual perceptual content can agree with these points while arguing that perceptual content is conceptual after all. One way to do so is to draw a distinction between absolute and relative nonconceptual content, where absolute nonconceptual content involves a difference in kind from that of propositionally structured thought, and relative nonconceptual content does not. Jeff Speaks elucidates relative nonconceptual content as follows.

A mental state of an agent A (at a time t) has relatively nonconceptual content iff the content of that mental state includes contents not grasped (possessed) by A at t. (2005, 360)

This distinction is open to me in the present context. I can claim that motor representations do not take a fundamentally different kind of content. Rather, the content of motor representations is relatively nonconceptual—an agent can token a motor representation M even though she does not grasp concepts included in M's content.

What would it mean to claim that motor representations possess conceptual content? It is necessary (though insufficient) that they possess contents with a compositional structure. Some would maintain that the kind of compositional structure required is that of predicate logic. But some philosophers have argued that a kind of weak systematicity that falls short of fully logical structure could be sufficient for concept possession (Carruthers 2009).

Consider, for example, analogue magnitude representations: “primitive representations of spatial, temporal, numerical, and related magnitudes” (Beck 2015, 830). Beck (2014, 2015) notes that analogue magnitude representations follow Weber's Law—“as the ratio of two magnitudes approaches 1:1 they become harder to discriminate and beyond a certain threshold… they cannot be discriminated at all” (2015, 833). As a result, analogue magnitude representations fall short of logical structure. To take Beck's example, one could use analogue magnitude representations to judge that 9 is less than 18, and to judge that 10 is less than 20, while lacking the representational competence to judge that 9 is less than 10. If one thinks (as Beck does) that conceptual content requires fully general systematicity, this would indicate that analogue magnitude representations have non‐conceptual content. But if one only requires that a state's content be apt for use in productive forms of reasoning, analogue magnitude representations could qualify as conceptual. This is because they possess some structure, and as such appear to be useful in practical reasoning (see Beck 2015).

Now, motor representations possess some structure. That they do so is central to their playing important roles in sensorimotor control and learning. Motor representations specify ways of moving apt both for generating predictions about upcoming sensory consequences, and for construction of ever more sophisticated motor schemata via learning processes of ‘chunking’ and ‘parsing’ (Graybiel 1998).^8^

Suppose one thought that this structure was not sufficient to attribute conceptual content to motor representations. One could then take a different cue from debates over perceptual content (see, e.g., Evans 1982, 227), arguing that the uptake of motor representations into propositionally structured thought involves conceptualization. If agents are able to conceptualize elements within their accessible motor representations—for example, movement profiles, amounts of effort involved to move in certain ways, relationships of compatibility and incompatibility between discrete patterns of movement—they could then link action concepts to motor representations within explicit practical reasoning, utilizing the latter within practical reasoning as time and reasoning abilities allow.

But how would agents carry out the translation process involved in conceptualization? Consider that on a plausible picture of human cognitive architecture, adult human cognitive sophistication is built upon a scaffolding of modules that operate on representational states formatted in a range of ways. Penn et al. (2008) have argued that one important difference between human and non‐human animals is that in addition to modules subserving various areas of cognitive and perceptual competence, humans “possess the additional capability of reinterpreting these perceptually grounded representations in terms of higher‐order, role‐governed, inferentially systematic, explicitly structural relations” (Penn et al. 2008, 127). In the present context, the role of reinterpretation is key. Recent discussion of representational kinds has illuminated, for example, cartographic representational formats (Rescorla 2009), analogue magnitude representational formats (Beck 2015), and iconic representational formats (Carey 2009), among others. How on earth could we manage to bring contents in diverse formats together in productive forms of thought and reasoning? Notice that we are no longer asking a question about a problem unique to action control. We are asking a question fundamental to cognitive science: this version of the interface problem is everyone's problem.

This is not the place for a full discussion of ways to think about this issue, but one influential recent proposal due to Susan Carey involves reference to Quinean bootstrapping. This is a process of conceptual construction and change that draws heavily on, as Carey explains, “explicit symbolic representations to formulate placeholder structures and on modeling devices such as analogy, thought experiments, limiting case analyses, and inductive inference to infuse the placeholder structures with meaning” (Carey 2011, 121). Through bootstrapping the cognitive system develops richer and more powerful bodies of knowledge on the back of earlier and more primitive structures. It may be that this is how human agents move from the primitive ‘motor vocabularies’ (see Rizolatti et al. 1988) given at birth to the wide array of action‐types development of language and inculcation in society makes possible.

Whether Carey is right or not (for relevant discussion, see Shea 2011), the present point is that if the coherent utilization of multiple representational formats within practical deliberation involves a translation process, we can understand this translation process as structured by the kind of learning that generates our sophisticated, propositional‐level action concepts in the first place. So this is not a process about which we know nothing at all, even if the details remain far from perfectly understood.

In this connection, a further (somewhat speculative) point may be helpful as a spur to future work. It seems plausible to me that proximal intentions—intentions to A now—regarding unfamiliar actions will require motorically (and sensory‐motorically) formatted contents, and that as one becomes more familiar with the performance of an action, one will gradually acquire and develop connections to propositionally structured reasoning. I envision this process happening for adult agents tasked with learning unfamiliar action‐types, but we can consider the point as applied to young children as well. Consider the fact that children between one and two years of age are able to control their own actions to some degree, well before propositionally structured reasoning is fully developed. Children at this age are also able to understand what other agents are trying or intending to do enough to provide instrumental help in at least some conditions. For example, in an experimental condition that had an experimenter bump into closed cabinet doors with a stack of magazines, 18‐month‐olds tended to help the experimenter by opening the cabinet doors (Warnecken and Tomasello 2006). And when an experimenter is using an item such as a clothespin and drops it out of reach, many 18‐month‐olds (and a higher proportion of 30‐month‐olds) will pick the item up and return it immediately, even without receiving cues or signals from the experimenter that this is what is desired (Svetlova, Nichols, and Brownell 2010). Interestingly, 14‐month‐olds will also provide help in such conditions, and can distinguish between conditions when the experimenter needs help and conditions in which the experimenter intentionally throws the clothespin on the floor. But unlike 18‐month‐olds, 14‐month‐olds do not appear able to provide help for instrumentally more complex actions. For example, 14‐month‐olds do not appropriately help an experimenter when she bumps into closed cabinet doors, nor when she attempts to reach a lost spoon through a flap that is too small for her hand (Warnecken and Tomasello 2007).

How is this work relevant to our present concerns? These studies indicate that agents as young as 14 months old have a developing understanding of the structure of action and the relationship between means and ends. (These studies also indicate that their understanding of this structure improves as they age, with improvements in instrumental helping noted at 18 months, 24 months, and 30 months.) What kinds of mental states and processes subserve this understanding? I submit that whatever our answer, it will not include exclusively propositionally structured states and reasoning processes. This has implications for how we think of the development of intentions. Plausibly, our first steps towards sophisticated practical reasoning involve thinking about how movement profiles can be combined to constitute achievable action plans for action‐types of varying degrees of complexity. If so, we have reason to think that intentions are not exclusively structured as attitudes to propositions, but rather that intentions can be attitudes or commitments regarding movement profiles and goals, which can be represented in the kinds of formats available to a 14‐month‐old.

Stepping back now, readers will hopefully have noted that my proposal takes on board elements from both Butterfill and Sinigaglia and Mylopoulos and Pacherie. I think Butterfill and Sinigaglia are right to highlight the importance of a deferral process for some instances of action control. But I agree with Mylopoulos and Pacherie that this process alone seems inadequate to explain the relevant phenomena. In particular, their appeal to deferral seems to need an explanation of how agents manage to translate or otherwise connect motor representations and intentions. In this connection, I think Mylopoulos and Pacherie are right to highlight the importance of learning in generating motor schemata and action concepts. I think that learning and processes of conceptualization may be the processes that enable an agent to fluently and flexibly move between states with different representational formats. I found Mylopoulos and Pacherie's account of the link between motor schemata and action concepts unsatisfying, however, and sought to push beyond it in a few crucial ways. I emphasized the location of the relevant learning processes in a more general problematic regarding the ways agents acquire the capacity to work with multiple representational formats. I also rejected the idea that motor representations are inaccessible to consciousness, and emphasized an agent's ability to put propositional level action understanding and (some aspects of) motoric level action implementation together within explicit practical reasoning—a point that Mylopoulos and Pacherie would likely reject, but that I find potentially very important. Elaborating upon this latter claim, I suggested that agents may begin to gain practical reasoning abilities with respect to action plans represented in sensorimotor formats, and move from this to propositional thought about action plans as the action plans become more familiar, and more closely connected to related items such as nearby action‐types, and the agent's abilities.

What is the upshot of this discussion? By placing the interface between propositionally structured thought and motor representation within explicit practical reasoning and intention formation, we acquire tools useful for thinking about how the interface is bridged. Although we fail to perfectly understand the process, at least two options are available for further explicating my proposal. On the first, motor representations do not possess a fundamentally different *kind* of content from propositionally structured thought. Rather, since motor representations possess conceptual structure they can enter into practical reasoning in ways this structure allows. On the second, motor representations possess nonconceptual content, but agents possess the capacity to conceptualize information motor representations contain—for example, movement profiles, amounts of effort involved to move in certain ways, relationships of compatibility and incompatibility between discrete patterns of movement, and so on. It remains to explain how this conceptualization process works and what kinds of practical reasoning conceptualized motor representation makes possible. But these are not processes about which we know nothing.^8^

## 6 Conclusion and Implications

I conclude that the interface problem can be solved by placing the interface within explicit practical reasoning. Agents have the capacity to specify motoric parameters for action execution at the personal level, and when they do so their intentions can lead a double life, taking both propositionally and motorically formatted contents.

In this paper's introduction I noted a solution to the interface problem would likely have downstream consequences for theories of skilled action and its relation to knowledge. Here I focus on recent claims about the intelligence of motoric‐level action implementation processes. I avoid nearby claims about the knowledge‐involvement and practical rationality of such processes, since they seem to me to involve a wider range of ancillary philosophical issues, and a paper's conclusion is not the right place for such a discussion.

What is it for a process or family of processes to be intelligent? Neil Levy offers a plausible stipulation: “The genuine mark of intelligence, I claim, is the capacity to flexibly adapt in an appropriate manner to environmental perturbations” (2017, 317). Let us take this stipulation on board. According to the standard it sets, it is clear that motoric‐level processing possesses a measure of intelligence. Motoric‐level processes implement intentions in a way that is sensitive to task‐demands, to ongoing perturbations, and to the demands of skill learning quite generally. But to what degree are these processes intelligent? The picture offered in section 3 suggests that, independently of intentions, such intelligence is limited. Motoric‐level processes are sensitive to *sensory* feedback, and make adjustments accordingly. But as McDougle et al. note, “implicit recalibration is completely insensitive to task success” (2016, 539). This suggests that the much broader intelligence of skilled action consists largely in the contributions of higher‐level action understanding. As emphasized above, agents are able to make an explicit contribution to action control such that they override implicit learning to achieve explicitly represented goals.

One might think my emphasis on the contribution of explicit cognition to intelligent action runs counter to Fridland's (2017) recent argument that motor control is intelligent “all the way down.” But I think our disagreement is minor. Fridland rejects five claims regarding motoric‐level action implementation: that it operates ballistically, that it operates invariantly, that it operates independently, that motor processes “blindly implement some general, pre‐planned trajectory” (1541), that they are insensitive to the semantic content of personal‐level goals, and that they are independent of intentional states (like intentions). Notice that rejection of these last two claims is required by my own proposal. Further, I share Fridland's rejection of the first three.

Nonetheless, there is a minor disagreement, and it may have important implications for philosophical accounts of skilled action. Consider Fridland's defense of the claim that “the detailed kinematic strategies executed in motor skills do not blindly implement some general, pre‐planned trajectory but, rather, unfold in an intelligent way” (2017, 1540). As partial support Fridland discusses a study by Liu and Todorov (2007). This study involved reaching to targets with late perturbations introduced, and it has been influential in confirming *optimal control theory*—a rich model of the surprisingly complex dynamics within motoric‐level adaptation processes. Fridland interprets that study as demonstrating the following.

[Liu and Todorov] are able to show not only that certain perturbations remain uncorrected but that these perturbations remain uncorrected because they are irrelevant for task success and not simply because there is no time to correct them. This finding supports the notion that fine‐grained sensorimotor control is flexible insofar as corrections are made in an intelligent way – not simply to conform to a pre‐determined trajectory, but in order to achieve one's goal. That is, if a correction is unnecessary for task‐success, even if it was part of an original motor plan, after perturbation, it remains uncorrected. (1541)

There is an important equivocation regarding task‐success in Fridland's interpretation that threatens to give motoric‐level action implementation too much credit. What Liu and Todorov showed was that late perturbations—the movement of a target after a reach towards the target had already begun—lead to undershooting of the target even when there is time to correct for it. So if we take task‐success to mean perfect satisfaction of an intention, Liu and Todorov did not show that sensorimotor processing ignores perturbations to achieve task‐success.

However, the sensorimotor control system's operations regarding undershooting can be explained in a way that renders them sensible. One major achievement of the Liu and Todorov paper was that they developed a model that explained this puzzling result. The model posited that sensorimotor computations are sensitive to a trade‐off between keeping the hand stable after it hits the target and accuracy at hitting the target. Confirmation of the model occurred when Liu and Todorov gave one set of participants the explicit instruction to stop their hand at the target, and another set of participants no such instruction. Participants in the latter set showed higher arm velocity and showed greater accuracy, confirming the prediction that manipulating the stability‐accuracy trade‐off would lead to different sensorimotor implementation.

Importantly, however, in light of the double life of intention I have emphasized in this paper, it is difficult to interpret this result as the sole achievement of ‘intelligent’ motoric‐level action‐implementation. It seems, rather, that the differing sets of subjects possessed different intentions—one set intended to bring the hand to a stop before hitting the target. The sensorimotor implementation processes displayed sensitivity to these different intentions, but transformed them according to their own principles that, in this case, involved a trade‐off between stability and accuracy, leading to less accuracy in the task when stability was given more weight. Seen from a certain height, the moral of the story is similar to that of section 3. Motoric‐level action implementation has a role to play—usually to the benefit of skill learning, but sometimes to the detriment of perfect intention satisfaction. And action plans specified by explicit intentions—plans that can take motorically formatted contents—are crucial for explaining how the action is guided.^9^

The upshot is that the bulk of the intelligence displayed by skilled action is the agent's intelligence, as embodied and expressed in the explicit practical reasoning processes where propositionally structured thought and motorically formatted goals interface.

## Notes

2 A referee notes that Mylopoulos and Pacherie could avoid the translation process worry if they denied that action concepts are personal‐level objects available to be slotted into propositions. But the referee also notes, rightly in my view, that pushing action concepts to a level beneath proposition‐level action understanding leaves mysterious how agents could think about their actions using action concepts.

3 For additional criticisms of the views considered in this section, and for an interesting positive proposal, see Burnston (2017). Burnston's paper was published just as this paper was accepted: I hope to engage more substantively with it in future work.

4 A number of authors (e.g., Rizolatti et al. 1988, Butterfill and Sinigaglia 2014) argue that motor representations do not simply represent kinematic movement profiles, but represent action goals. A referee suggests that if the propositional level and the motoric level represent a similar goal in different ways, this may explain the results in a way consistent with Butterfill and Sinigaglia's deference model. I do not think however, that the fact that motor representations represent action goals in a different way can help here. The point is that agents do not simply defer to their motor representations, they explicitly create goals in order to override what they take their motoric level processes to be doing.

5 At this juncture, a referee raises an interesting point. The studies at issue direct focus to the fact that intentions and motor representations represent an action's end‐state or goal. But it is plausible that the mental states guiding skilled action represent not just goals, but also means to achieve the goals. Accounting for how intentions may include both propositionally and motorically formatted representations of means is somewhat beyond the scope of this paper, but in my view Sinigaglia and Butterfill's (2015) discussion of the use of imagery offers interesting material for reflection. In particular, in thinking about how an agent manages to actively guide the way that she achieves some goal, I would emphasize an agent's ability to combine perceptual, motoric, and proprioceptive imagery into coherent movement profiles that could set parameters for action guidance and the detection of error at relatively fine‐grained levels.

6 Although she says nothing about intentions or motor representations, Carlotta Pavese (2015) argues that the ability to A intentionally requires knowledge of a rule to A, and that a rule to A is a matter of possessing a practical concept that could serve as a “conceptual component of propositions that can be known” (166). If intentions correspond to propositions, and (at least some) motor representations to practical concepts, Pavese's view might be seen as a way of fleshing out my claims here.

7 For a different (but I think ultimately congenial) discussion of a role for consciousness in solving the interface problem, see Sinigaglia and Butterfill (2015).

8 Those steeped in this literature may wonder whether there is a conflict between my proposal and a well‐known result due to Anthony Marcel and colleagues. In that study (reported in Marcel 2003), experimenters used vibro‐tactile stimulation to the elbow to generate an illusory experience in participants that their arm was located to one side of an action target, when in fact their arm was located on the other side of the action target. When experimenters had participants reach for the target, they were able to do so successfully, even though 60‐70 percent of participants gave no indication they were aware of having moved in a direction opposite to their consciously reported intention. Furthermore, 30‐40 percent of participants did report an awareness of conflict. Marcel reports that “All of the latter subjects commented in one or another way that there was something peculiar, or that while they had intended to move in one direction they had the impression that they had moved in the other” (2003, 66). So this looks like a case in which specification of the motor plan overruled the conscious intention. Is this not in tension with my proposal involving conscious intentions specifying motor representations? In response, I think it is important to observe that this is a case of object‐directed action, for which visual feedback is crucial. Participants were told to reach towards a target that they could see. It is possible that while agents can in some circumstances override implicit learning to ensure action success, object‐directed actions cede control to guiding visual representations. It is well‐known that small deviations from an action plan are often corrected in accord with visual feedback without the agent's being aware of the correction (Fourneret and Jeannerod 1998). And there are many familiar actions—e.g., catching a baseball—for which visual feedback appears to play the primary guiding role. Indeed, perform the experiment on yourself by having someone throw a ball to unpredictable locations near your body. Do you experience yourself having fine‐grained control over ball catching? This can be quite uncanny but I have the experience of my arm and hand working more or less on their own to ensure that I catch the ball, just ahead of what I consciously realize I am doing. Ultimately, then, I do not think this kind of result is in conflict with my proposal. What this kind of result demonstrates is that there is a back and forth between proposition‐level action understanding and motoric‐level action implementation, and that in some cases—for good pragmatic reasons—sensorimotor feedback loops gain the upper hand. This is consistent with the conscious intention playing a motorically guiding role in a wide range of cases.

9 This is consistent with Liu and Todorov's interpretation of their main result: “changes in stopping requirements … caused changes in the way visual feedback is used to make on‐line corrections. This may be the first demonstration that visuomotor feedback loops are affected by the task and in particular by nonvisual components of the task” (9367).
